# Taguchi-based optimisation of FSW parameters for advancement in aerospace materials: Al-Li 2060 alloy

**DOI:** 10.1016/j.heliyon.2024.e41048

**Published:** 2024-12-07

**Authors:** Noah E. El-Zathry, Stephen Akinlabi, Wai Lok Woo, Vivek Patel, Rasheedat M. Mahamood

**Affiliations:** a Department of Mechanical and Construction Engineering, Northumbria University, Newcastle Upon Tyne, NE1 8ST, United Kingdom; b Mechanical Engineering Department, Benha University, Benha, Egypt; c Department of Engineering Science, University West, Trollhattan, 46186, Sweden

**Keywords:** Aluminium-lithium alloys, Friction stir welding, Microhardness, Microstructure, Axial force and taguchi method

## Abstract

Aluminium-lithium (Al-Li) 2060 alloy, a 3rd generation Al-Li alloy, is considered a structural material for aircraft components. This study employs the Friction Stir Welding (FSW) process with a kinematic 5-axis robotic arm to weld 4-mm-thick plates of 2060-T8E30 Al-Li alloy. The focus is on the impact of tool axial force and speeds on the microstructural evolution, mechanical properties, and surface integrity of the welded joints. The applied process parameters included rotational speeds ranging from 800 to 1600 rpm, traverse speeds from 2 to 4 mm/s, and axial forces from 4 to 6 kN. We utilise the Taguchi L9 orthogonal array to optimise the process parameters. The results revealed that rotational speed is paramount for affecting the welds' quality, followed by axial force and then traverse. Defect-free samples exhibited a fine surface finish, with average roughness values of 3.05 μm and 3.536 μm. The study also showed that 5 kN of axial force, 1200 rpm of rotational speed, and 3 mm/s of traverse speed were the best FSW conditions for getting a maximum stir zone microhardness value of 128.77 HV. This study also shows how to improve the FSW parameters for Al-Li alloys, showing how important precise parameter control is for improving joint strength and weld quality in high-tech aerospace and automotive applications.

## Highlights

• Investigating FSW axial force effects on 3rd gen Al-Li 2060 alloy.
• Optimizing FSW parameters, tool speeds, and axial force using the Taguchi method.
• Analysing hardness, microstructure, and surface roughness under various conditions.

## 1 Introduction

Aluminium-lithium (Al-Li) alloys are considered ideal engineering materials for various industrial applications, particularly aircraft components, due to their high strength-to-weight ratio compared to conventional aluminium alloys [1]. The third generation of Al-Li alloys, containing approximately 0.8 %–1.8 % lithium by weight, has overcome many issues associated with previous generations [2]. Among these, Al-Li 2060-T8E30 stands out as a third-generation alloy extensively used in aeronautical manufacturing due to its low density, high specific strength, and toughness [3]. Despite its benefits, Al-Li 2060-T8E30 alloy has problems when it is welded using standard fusion welding methods. These problems include the ability to form secondary and brittle phases, poor joint efficiency, and solidification cracking, which makes these methods less useful for this alloy [4]. These issues have been successfully addressed using the Friction Stir Welding (FSW) process, which can also effectively avoid the loss of Li element, which participates in precipitation hardening [5].

The Welding Institute of the UK invented FSW, a solid-state welding process, almost 40 years ago for welding high-strength aluminium alloys [6]. Unlike traditional fusion welding methods, FSW does not necessitate the melting of the materials for joining. Instead, the process achieves bonding through frictional heat and plastic deformation [3]. Over time, FSW has expanded its applicability to include copper alloys, titanium alloys, and polymers [7,8]. This technique has proven effective in producing various welding configurations and has become the preferred method for aerospace applications [9].

**Fig. 1:**
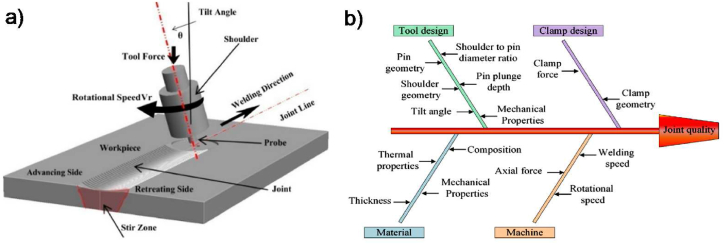
FSW process a) schematic diagram [3] and b) parameters [11].

The FSW process operates with a rotating tool consisting of a shoulder and a pin. This tool plunges into the joint line between workpieces until the shoulder contacts the upper surface. As the tool rotates, it generates frictional heat between the workpieces, softening the material beneath the shoulder to facilitate material flow around the pin. The tool then traverses along the welding line, transferring the plasticized material from the advancing side (AS) to the retreating side (RS), forming a solid-state joint. The four common welded zones in the FSW process are base metal (BM), stir zone (SZ), thermally mechanically affected zone (TMAZ), and heat-affected zone (HAZ) [10]. Fig. 1-a illustrates the schematic diagram of the FSW process.

Various FSW parameters co-determine welded joints' material flow and microstructure evolution. Fig. 1-b illustrates the different FSW process parameters. Among these, tool speeds (both rotational and traverse) are the most significant, as they primarily influence the welded joints' quality [11]. The thermal cycle and the heat generated during FSW are directly influenced by process parameters such as traverse speed and rotational speed. Studies have shown that reducing traverse speed while increasing rotational speed leads to grain growth and material softening, significantly affecting the microstructure of the weld [[12], [13], [14], [15]]. Tool geometry, including the design of the shoulder and pin, also plays a vital role in controlling heat generation and material flow. Shoulder geometry dictates the vertical compressive force and the generation of frictional heat, while pin geometry affects the SZ size and the effectiveness of material stirring. Variations in these geometric features directly impact the thermal cycle and the plastic deformation, both of which play a critical role in shaping the weld's microstructure. However, its influence on the overall joint quality is less significant when compared to tool speeds [[16], [17], [18]]. Additionally, parameters such as plunge depth, which dictates the tool's penetration into the workpiece, and tilt angle, the angle between the spindle axis and the workpiece, are critical in determining weld quality. These factors impact material mixing, weld penetration depth, and the weld zone's shape [[19], [20], [21]]. Furthermore, other studies have examined the influence of clamping conditions on the mechanical properties of friction stir welded joints [22].

FSW exhibits superior performance when welding Al-Li alloys compared to traditional welding techniques. Despite its numerous advantages, FSW does have some limitations. It may require specialised equipment and tooling. Moreover, there are constraints on the thickness of materials that can be effectively joined in a single pass. Also, certain defects, including hook defects, weld thinning, and flash defects, have been observed in Al-Li FSWed joints [23]. Consequently, researchers have sought enhancements to the FSW process. Nowadays, new welding techniques have been performed on Al-Li alloys, such as ultrasonic vibration-assisted FSW (UVaFSW) [24], friction stir additive manufacturing (FSAM) [25], heat backing plate assisting FSW (HBPaFSW) [26], underwater FSW [27], shot peening method assisted FSW [28], semi-stationary bobbin tool FSW (SSBFSW) [29], double-side pin-less friction stir spot welding (DPFSSW) [30,31], etc. These developed FSW techniques have notably minimised joint defects and enhanced overall joint performance.

Previous studies have examined how varying FSW process speeds affect the joint strength of traditional aluminium alloys. These studies reported that the tool rotational speed does not impact joint strength significantly, whereas the traverse speed has a more pronounced effect, according to Refs. [32,33]. However, Mao et al. [34], Yan et al. [35], and Liu et al. [36] saw that the joint strength of FSWed Al-Li 2060–T8 first got stronger and then got weaker as the traverse or rotational speed went up. Table 1 presents the utilised welding speeds. Meanwhile, Tao et al. [37] observed a decrease in the joint strength of the FSWed Al-Li 2198-T8 alloy when they increased the tool's rotational speed from 800 rpm to 1600 rpm, while maintaining the traverse speed at 200 mm/min. Ma et al. [38] investigated the FSW joint strength of Al-Li 2060-T8 alloy under two cooling conditions: natural cooling and water cooling, both at 800 rpm–200 mm/min. They observed a "W"-shaped microhardness distribution, with the minimum microhardness located at the boundary between HAZ and the TMAZ on the RS. For water cooling, there was a slight difference of 3.2 HV compared to natural cooling. However, there is a lack of detailed explanation regarding the mechanism that governs the relationship between joint strength in FSW, process speeds, and axial force.

**Table 1:** Summary of FSW research work on Al-Li 2060-T8 joint.

Al-Li alloy	Investigated parameters			Investigated characteristics	Ref
	Rotational speed (rpm)	Traverse speed (mm/min)	Axial force (kN)		
2060-T8	750–1500	95–150	–	Joint formation and mechanical properties.	[34]
2060-T8	400–1300	100	–	Mechanical properties and joint characteristics	[35]
2060-T8	600–1000	300	–	Mechanical properties and the development of microstructural precipitate.	[36]
2060-T8	800	200	–	Effect of cooling conditions on joint formation and mechanical properties.	[38]
2060-T8	2400	100	–	Impact of in-process water cooling on joint characteristics	[39]
2060-T8	2500 rpm	–	5.1–5.8	Joint formation and mechanical properties of friction stir spot welded joints	[42]
2060-T8	400–1200	50 and 200	–	Hardness, tensile strength, and microstructural precipitate development	[43]

In other research, the effect of precipitation on the tensile properties of the third generation of al-Li alloys was looked at in the FSW zone. The third generation of Al-Li alloys, including 2060-T8, exhibits unique characteristics in microstructural evolution and mechanical properties compared to conventional aluminium alloys. Researchers have found that different FSW process conditions have different precipitates in the SZ that have a big impact on the strength of the joints [[39], [40], [41]]. These precipitates are called T (Al20Cu2Mn3), T1 (Al2CuLi), ε′ (Al3Li) + β′ (Al3Zr) or T1 + S′ (Al2CuMg). Qin et al. [40] studied the precipitation distribution in the 2195-T8 Al-Li alloy FSW joint at 600 rpm and 200 mm/min, finding the HAZ to be the softest region. This was attributed to the T1 and θ′ precipitate dissolution. Therefore, we can conclude that the welding parameters and the welded materials determine the precipitate distribution. The redistribution of precipitates was similar, as seen by Cai et al. [39]. The T1, θ′, and S′ phases made the BM stronger, while the precipitates broke down in the SZ and TMAZ. They attributed the variation in hardness within the weld zones to the precipitates' dissolution and coarsening sequences. Gao et al. [41] reported comparable findings, linking hardness variations to precipitate coarsening and dissolution.

Despite the advancements in FSW of Al-Li 2060 alloys, there is still a lack of clarity in the current literature, particularly regarding the impact of axial force on joint quality. Table 1 underscores this gap by summarising the parameters investigated for FSWed Al-Li 2060-T8 joints. Therefore, further investigation is crucial to clarify the impact of axial force and its interaction with tool speeds, providing more definitive insights.

This paper aims to optimise parameters, including axial force and tool speeds, to demonstrate the effects of varying these parameters on joint quality. The quality of the welded joints was evaluated through microhardness measurements and microstructural analysis using optical microscopy (OM) in various welding conditions.

## 2 Materials and methods

**Fig. 2:**
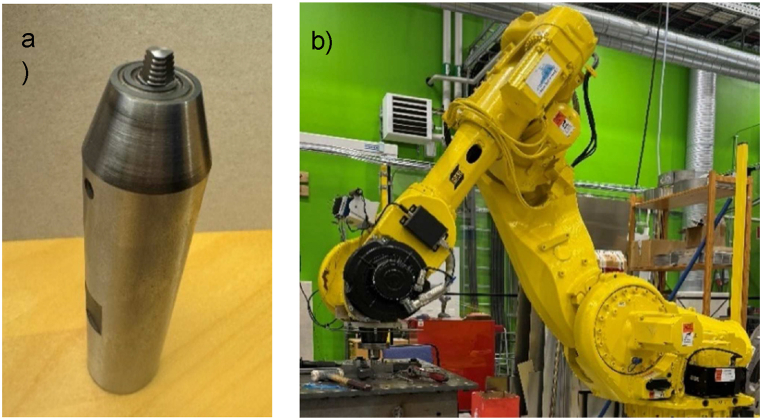
a) FSW tool process and b) FSW robot arm (at the PTC in Trollhättan, Sweden).

In this experiment, we welded 4.0 mm thick 2060-T8E30 Al-Li plates using a hot-work tool steel (H13) with a 12 mm shoulder diameter. The tool featured a scroll-designed shoulder and a tri-flats threaded pin with a 6 mm root diameter and a 4 mm tip diameter, as shown in Fig. 2-a. These tool features were selected based on their demonstrated high performance in previous studies [[44], [45], [46]]. Throughout the experimental runs, the tilt angle was consistently maintained at 1°, following the methodology established in previous work by Ref. [47]. The Production Technology Center (PTC) in Trollhättan, Sweden, used a kinematic 5-axis robotic arm system with a control system to conduct the welding process. The design matrix guided the execution of the welding operations in a randomised sequence. Fig. 2-b shows the FSW process setup. We use brief forms to designate the welded samples. For instance, sample 1200-2-5 denotes the welding conditions of 1200 rpm, 2 mm/s, and 5 kN.

For metallurgical analysis, microstructure and microhardness specimens of 20 mm × 4 mm were taken along the welding direction according to ASTM standard E3-11. These specimens were polished following traditional metallographic procedures and then etched using Keller's reagent for 10–20 s. The microstructural analysis was conducted using an optical microscope (Zeiss Axio, Carl Zeiss AG, Germany). The microhardness values of SZ, TMAZ and HAZ were measured along the joint centerline using a Shimadzu HMV Microhardness Tester (Shimadzu Corporation, Japan) at the Production Technology Center (PTC) in Trollhättan, Sweden. A diamond indenter was applied with a force of 1.961 N for a dwell time of 10 s, with measurements taken at 0.5 mm intervals. The average hardness values of the SZ for each sample were calculated and utilised to optimise the process parameters.

### 2.1 Design of experiments

In this investigation, we applied the L9 orthogonal array designed by the Taguchi method, incorporating three process parameters: axial force and tool speeds (rotational and traverse). Although various statistical methods, such as Response Surface Methodology (RSM) [48] Analysis of Variance (ANOVA) [9], and Grey Relational Analysis (GRA) [49], have been successfully applied in previous studies to optimise process parameters; the Taguchi method was chosen for this study due to its ability to achieve optimisation with a limited number of experiments. In the context of FSW, where multiple variables can affect outcomes, the Taguchi method offers a robust experimental design with fewer trials compared to methods like RSM. This makes it suitable for our study.

Table 2 presents the specific process parameters and their corresponding levels. The utilised range of the tool speeds was selected based on the literature [34,36,43], while the range of axial force was determined through trial experiments, as there have been fewer studies investigating its effect on the FSW of Al-Li 2060-T8 plates. In these trials, we maintained a constant rotational speed of 1200 rpm and a traverse speed of 3 mm/s, which were established as intermediate levels for the input parameters. We then varied the axial force to assess its impact on the welding process. These experiments demonstrated that when the axial force was below 4 kN, defects such as incomplete penetration occurred. Conversely, at forces exceeding 6 kN, issues like excessive flash and thinning of the weld were observed. Based on these findings, we established the optimal axial force range to be between 4 kN and 6 kN, which effectively balances defect formation and ensures sound weld quality. This approach allowed us to optimise the welding parameters for the material and thickness utilised in our study.

**Table 2:** FSW process parameters.

parameters	Symbol	Units	Levels		
			1	2	3
Rotational speed	TRS	rpm	800	1200	1600
Traverse Speed	TTS	mm/s	2	3	4
Axial force	AF	kN	4	5	6

The Taguchi technique used the signal-to-noise (S/N) ratio objective function to optimise the process parameters. The Taguchi method uses three categories to calculate the S/N ratio: nominal, larger, and smaller. We identify the optimal conditions for process parameters by evaluating the S/N ratio in relation to these categories [50]. This study employs the larger-the-better category to calculate the S/N ratio. This criterion focuses on maximising the desired output, ensuring that higher values of the output parameter lead to better process performance.

(1) $$Larger\;the\;betterS/N\left(db\right)=-10\times \log\;10\left(\frac{1}{n}\sum_{i=1}^{n}\frac{1}{y_{i}^{2}}\right)$$

where n represents the evaluation number and $y_{i}$ denotes the observation result for the $i^{th}$ performance characteristic.

## 3 Evaluation of experimental results

### 3.1 S/N ratio evaluation

We used MINITAB 15 software to analyse the experimental data and calculate the S/N ratio for each level of processing conditions using Eq. (1). The S/N ratio helps to evaluate the robustness of the process, with higher values indicating better performance relative to noise factors. Table 3 displays the FSW experimental runs and outputs, while Table 4 provides the estimated S/N ratio values. A higher S/N ratio value indicates that the process parameters are closer to their optimal levels, improving the overall quality of the weld.

**Table 3:** FSW experimental runs.

No.	TRS (rpm)	TTS (mm/s)	AF (kN)	Microhardness at the SZ (HV)
1	800	2	4	119.932
2	800	3	5	125.273
3	800	4	6	122.082
4	1200	2	5	127.827
5	1200	3	6	125.099
6	1200	4	4	123.303
7	1600	2	6	116.095
8	1600	3	4	118.593
9	1600	4	5	120.455

**Table 4:** Responses values for means and S/N ratio.

Level	Response for Means			Responses for S/N Ratios		
	TRS	TTS	AF	TRS	TTS	AF
1	122.4	121.3	120.6	41.76	41.67	41.63
2	125.4	123.0	124.5	41.97	41.79	41.90
3	118.4	121.9	121.1	41.46	41.72	41.66
Delta	7.0	1.7	3.9	0.50	0.13	0.28
Rank	1	3	2	1	3	2

To determine the significance of each process parameter on the microhardness of the welded joints, we ranked them based on the variation in delta statistics (the difference between the highest and lowest S/N ratios for each parameter). The larger the delta value, the greater the influence of the parameter on the response. As shown in Table 4, we ranked the process parameters in decreasing order of their impact: rotational speed, traverse speed, and axial force. This suggests that rotational speed is the most influential factor, playing a critical role in determining the microhardness, followed by axial force, which controls the amount of heat generation. Traverse speed, while important, showed a comparatively lower influence in this case. These findings highlight the importance of fine-tuning rotational speed and axial force for optimizing the mechanical properties of friction stir welded joints.

### 3.2 Analysis of Variance

Analysis of Variance (ANOVA) is a statistical technique widely used to evaluate the impact of input parameters and their interactions on process outputs, making it a valuable tool for parameter optimisation. ANOVA also ranks the parameters by their contributions to the total variation observed in the outputs, allowing for a clear understanding of which factors most influence the process. This ranking provides the contribution percentage of each process parameter, helping to identify the most critical factors in achieving optimal results [50].

Table 5 presents the contribution percentages of tool rotational speed (TRS), tool traverse speed (TTS), and axial force (AF) to the microhardness of the welded joints. The results indicate that all three parameters play a role in determining the microhardness. However, TRS stands out as the most significant parameter, accounting for 68.46 % of the total variation. This highlights the crucial role that rotational speed plays in controlling heat generation and material flow during the welding process. AF follows with a contribution of 24.99 %, indicating its importance in applying sufficient pressure to forge the materials together. Lastly, TTS exhibits the lowest contribution at 4.06 %, suggesting that while it influences the process, its effect on microhardness is relatively minor compared to the other parameters.

**Table 5:** Analysis of variance.

Source	DF	Seq SS	Contribution	Adj SS	Adj MS	F-Value	P-Value
Rotational speed (rpm)	2	74.670	68.46 %	74.670	37.335	27.50	0.035
Traverse Speed (mm/s)	2	4.425	4.06 %	4.425	2.212	1.63	0.380
Axial force (kN)	2	27.254	24.99 %	27.254	13.627	10.04	0.091
Error	2	2.715	2.49 %	2.715	1.358		
Total	8	109.064	100.00 %				

This ranking emphasizes the need to carefully control the TRS and AF to optimise the mechanical properties of the weld, particularly microhardness, while TTS can be adjusted with less impact on the outcome.

### 3.3 Development of regression model

A regression equation can be formulated to predict the microhardness of FSWed joints by analysing the relationship between FSW process parameters and the resulting mechanical properties. The regression model is expressed as a function of the input parameters:

Microhardness=f(TRS,TTS,AF)

The first-order polynomial equation (Eq. 2) represents this regression model, which is based on the regression analysis of the collected data.

(2) $$HV=122.073+0.356\;TRS_{800}+3.336\;TRS_{1200}-3.692\;TRS_{1600}-0.789\;TTS_{2}+0.915\;TTS_{3}-0.126\;TTS_{4}-1.464\;AF_{4}+2.445\;AF_{5}-0.981\;AF_{6}$$

The model's accuracy is evaluated using the determination coefficient, which measures how well the predicted values align with the actual experimental results. The developed regression model achieved a high determination coefficient, with R-Sq = 97.51 % and R-Sq(adj) = 90 %. These high values indicate that the model can predict the microhardness with a strong degree of accuracy, suggesting that the relationships between the input parameters and microhardness are well captured by the regression equation.

## 4 Results and discussion

Using suggested empirical relationships, we looked at how the FSW process parameters TRS, TTS, and AF affected the microhardness and surface roughness of surface composites in this study. Subsequent sections will investigate the influence of these parameters and discuss the underlying reasons for their effects.

### 4.1 Process parameters’ impact on microhardness

**Fig. 3:**
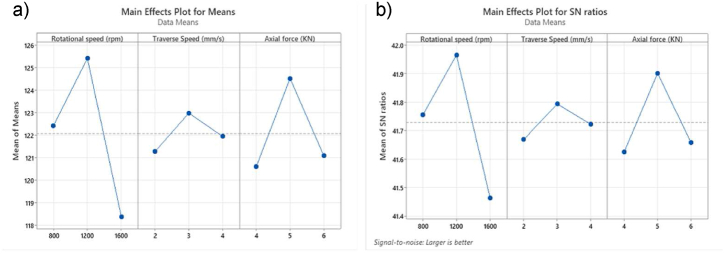
The main effect plot of microhardness is for a) means and b) SN ratio.

**Fig. 4:**
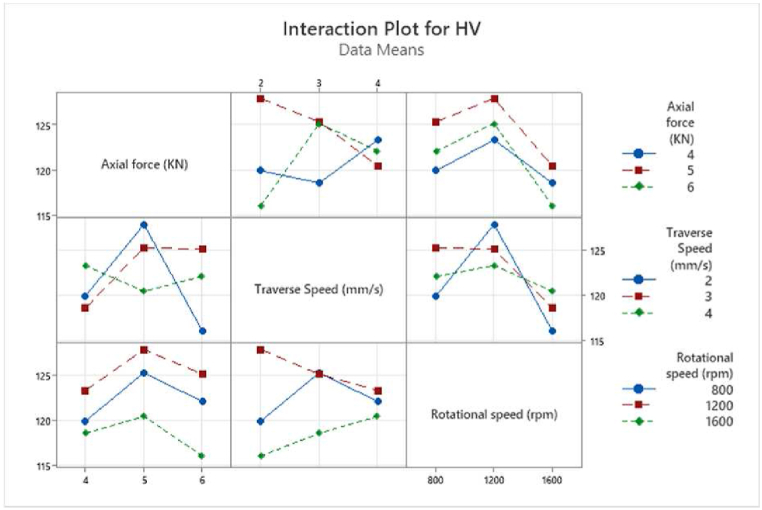
The interaction plot for microhardness.

The main effect plot illustrates the variation of process outputs with respect to the input variation. Fig. 3 illustrates the influence of input parameters on microhardness, with Fig. 3-a representing the mean values and Fig. 3-b depicting the S/N ratios. Additionally, Fig. 4 presents the interaction plot, showcasing the combined effects of welding parameters at various levels on the microhardness.

For rotational speed, the microhardness increases as the TRS rises from 800 rpm to 1200 rpm, reaching its maximum value before decreasing with further increases in TRS. The generated frictional heat and plastic deformation are impacted by TRS values. Insufficient heat is produced at low TRS and vice versa, which affects the mechanical properties. However, optimal TRS generates the optimal frictional heat, resulting in the appropriate stirring of the material around the pin. The drops in microhardness are attributed to the elevated peak temperature of the FSW thermal cycle, which promotes the formation of large, recrystallized grains, significant grain growth, and dissolution of precipitates [12].

A similar trend is observed for axial force, with the optimum value occurring at the middle level of 5 kN. The axial force has a significant impact on penetration depth and material flow during FSW process. Increased axial force increases penetration depth and contact area between the tool and workpiece, thereby significantly elevating frictional heat generation and influencing material flow dynamics. Adequate penetration ensures thorough bonding of the materials, thereby contributing to the strength of the joint. Furthermore, the formation of recrystallized grains and a fine-grained structure within the welded zone is facilitated by moderate axial force, leading to enhanced microhardness [51].

On the other hand, the effect of traverse speed on microhardness follows a similar trend to that observed with other parameters in this study, although it introduces slight variations in the microhardness distribution. This indicates that while traverse speed is an influential factor, its impact on the hardness profile may exhibit subtle differences compared to other welding parameters. The HV increases as TTS rises from 2 to 3 mm/s, followed by a slight decrease when TTS reaches 4 mm/s. The variation in hardness within the weld zone can be attributed to differences in heat generation associated with tool traverse speeds. At lower traverse speeds, the prolonged interaction between the tool and the workpiece results in higher heat generation, which leads to increased heat dissipation in the workpiece. This excessive heat promotes the formation of coarse grains in the weld zone, thereby reducing hardness. Conversely, higher traverse speeds decrease the interaction time between the tool and the workpiece, resulting in less heat generation [52].

**Fig. 5:**
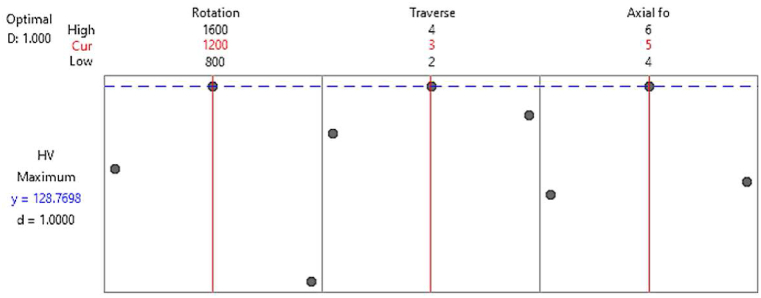
Optimal values of process parameters.

To achieve the highest microhardness value, the input parameters were optimised using ANOVA and regression modelling. The optimum level was determined to be 1200 rpm TRS, 3 mm/s TTS, and 5 kN AF to generate 128.77 HV based on the S/N ratio and Means data, as shown in Fig. 5.

### 4.2 Visual inspection

During the FSW process, several defects can be observed, including kissing bonds, tunnel defects, cracks, lack of bonding, cavities and voids. Hence, visual inspection of the weld surface quality is an important step to ensure the welded joint is free from macro defects.

**Fig. 6:**
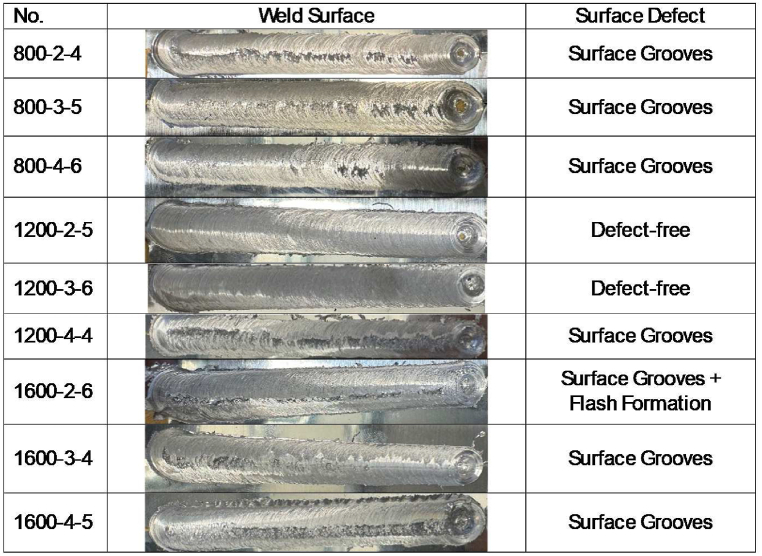
Weld surface information.

Following the welding operation, we visually inspected the samples and classified them as either defective or defect-free, as illustrated in Fig. 6. The high axial force causes surface defects in some samples, like the flash defects observed in sample 1600-2-6. Conversely, groove defects in samples like 800-2-4 and 1200-4-4 result from insufficient heat input. On the other hand, samples 1200-2-5 and 1200-3-6 had enough heat input to mix well in the SZ, cause plastic deformation, and allow material to flow, which resulted in joints that were free of defects. These findings underscore the importance of precise heat input control to achieve optimal weld quality and surface integrity.

### 4.3 Surface roughness

The amount of heat input plays a crucial role in determining the surface integrity of welded joints. The process parameters largely influence the achieved surface finish during FSW. Additionally, the feed marks formed by the FSW tool affect the stress concentration in the weld line [53].

The FSW process is distinct by a welding line with onion rings texture forms on the surface of the welding line. The height of this texture affects the overall topography and surface roughness, which in turn affects wear resistance and stress corrosion resistance [54]. The formation of this texture relies on the FSW tool movements: rotational and traverse in addition to plunge depth, which is analogous to axial force [55].

**Fig. 7:**
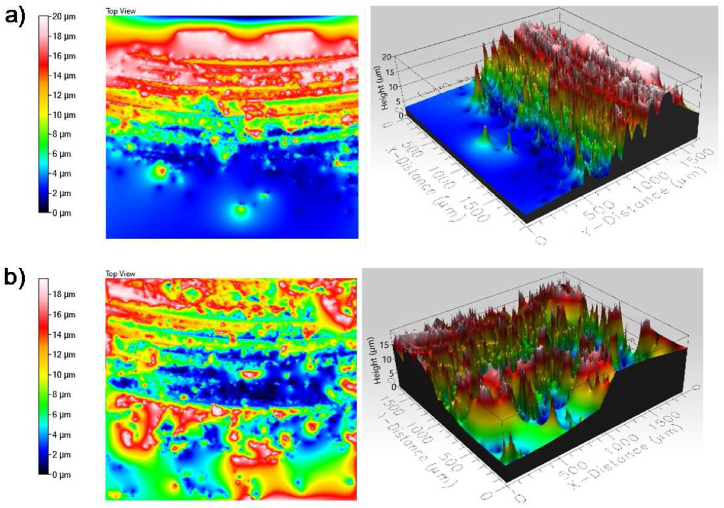
Surface topography and contour map for a)1200-2-5 and b)1200-3-6 samples.

We used a Profilm 3D optical profiler (Filmetrics Inc., USA) to examine the surface roughness of defect-free samples. Fig. 7-a and Fig. 7-b present the isometric surfaces and contour maps of samples 1200-2-5 and 1200-3-6, respectively. We took surface roughness measurements at five different locations along the welded line to ensure repeatability and obtain more valid results and recorded the average of these measurements. This study quantified surface roughness using the arithmetical mean roughness height (R_a_) parameter.

For the top surface of sample 1200-2-5, the average surface roughness was 3.05 μm, which is slightly higher than the 3.536 μm average surface roughness of sample 1200-3-6, indicating a smoother surface. as compared to Refs. [54,56]. This difference in surface roughness can be attributed to the thermal cycles experienced during the FSW process, highlighting the significant impact of process parameters on the overall topography.

Further improvement of surface integrity can be achieved through post-processing techniques such as deep rolling and post-weld heat treatment [57]. These techniques have shown promising results in enhancing the mechanical properties and overall quality of welded joints in FSW [58,59] and other welding techniques, such as laser welding [60,61].

### 4.4 Microhardness profile

**Fig. 8:**
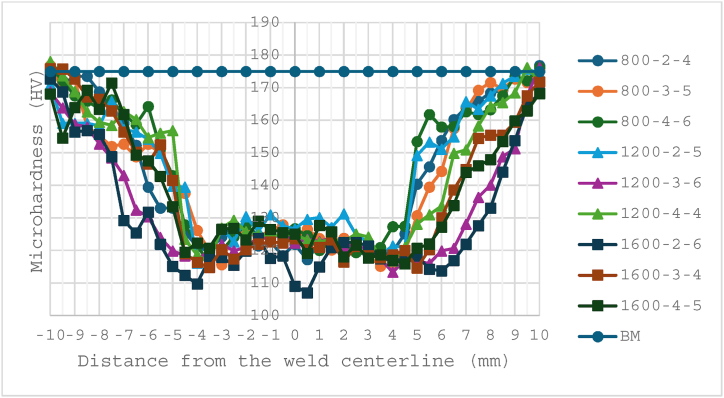
Microhardness distribution of the welded samples.

We cut the microhardness specimens into rectangular shapes (25 mm × 4 mm x 4 mm) perpendicular to the welding line and measured the microhardness along the mid-thickness of the cross-section. The BM's microhardness is around 175 HV, according to the testing results. We measured around 41 readings along the centerlines, with a 0.5 mm interval between two points. As shown in Fig. 8, all the hardness profiles are W-shaped. This is a common feature of high-strength, precipitation-hardened aluminium alloys when they are in the peak-aged temper condition [29].

In Al-Cu-Li alloys, the variation in microhardness between different FSW zones is influenced by the presence of precipitates and grain size, which are, in turn, affected by welding parameters. The thermal cycles in FSW cause recrystallization and grain growth, which directly affect the microhardness of the welded joints. Additionally, the grain structure of the material plays a critical role in determining microhardness by influencing deformation mechanisms such as dislocation motion and twinning [62,63].

During the thermo-mechanical welding process, precipitates undergo several transformations, including dissolution, coarsening, and re-precipitation of the strengthening precipitates. The BM has the highest number of precipitates, which significantly enhances its microhardness [43]. In contrast, the stir zone (SZ), characterized by finer equiaxed grains compared to the BM, could potentially exhibit increased hardness. However, the dissolution of most precipitates in the SZ counteracts the potential hardness increase from the finer grain size. Consequently, the microhardness of the BM is higher than that of the SZ, leading to joint failure typically occurring in the SZ [38].

The microhardness profile analysis reveals that the BM exhibits significantly higher hardness compared to the welded joints. The FSW process reduces the hardness of the weld region by approximately 30 %, resulting in an average hardness value between 116 and 128 HV. The microhardness distribution of all samples indicates that the HAZ and SZ have lower hardness values, which aligns with findings from previous studies [35,36,39,43]. The lowest hardness is observed near the interface between the TMAZ and HAZ, with values ranging between 110 and 115 HV at various locations. Overall, the microhardness in the SZ, HAZ, and TMAZ is lower than in the BM, which exhibits hardness over 175 HV.

Among the FSWed samples, the lowest microhardness was observed in samples 1600-2-6 and 1600-3-4, with microhardness in the SZ ranging from 107 to 123 HV. This can be attributed to the rotational speed of 1600 rpm, which generates high frictional heat, and the axial force of 4 kN, which is insufficient to achieve the required welding condition for Al-Li 2060, resulting in decreased microhardness values in the welding zones.

Conversely, the highest microhardness was noted in samples 1200-2-5 and 800-3-5, with microhardness in the SZ ranging from 120 to 131 HV. Despite the low rotational speed in sample 800-3-5, the hardness value remained high, likely due to the optimal axial force value of 5 kN. The highest microhardness value was measured in sample 1200-2-5, attributed to sufficient heat generation that enhanced material flow and produced an SZ hardness of about 130 HV. The reason for the SZ exhibiting higher hardness may be a uniform distribution of strengthening precipitates and the formation of the intermetallic compound. Hence, Optimal welding parameters, such as sufficient axial force and appropriate tool speeds, are crucial for achieving higher microhardness in the welded zones.

### 4.5 Metallography and microstructure evolution

**Fig. 9:**
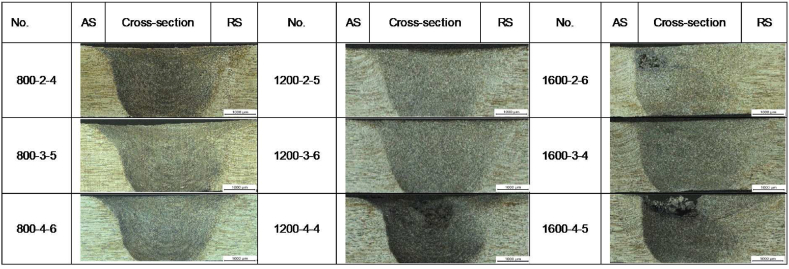
Macrostructures of samples at different welding conditions.

We used an optical microscope to obtain the macrostructure of Al-Li 2060-T8E30 FSWed samples, as illustrated in Fig. 9. The AS and RS are located on the left and right sides of Fig. 9, respectively. The four common welded zones are clearly observed in these cross-sections. The observed variation in grain size and structure of these zones is due to variations in heat inputs and material flow.

Fig. 9 clearly demonstrates that the SZ/TMAZ interface is more prominent on the AS than on the RS, with the TMAZ extending to the SZ on the RS, as seen in samples 800-3-5, 1200- 4-4, 1600-2-6, and 1600-4-5. The dynamics of the material flow also contribute to this extension, as observed in Ref. [36], which can be attributed to the strain induced by the stirring action of the tool pin during welding. The tool shoulder primarily influences the material flow dynamics, significantly contributing to heat generation. This heat softens and reduces the flow stress of the material beneath the shoulder, making it easier to deform and move. The shoulder's rotational movement propels this softened material, creating a rotational flow pattern. Consequently, the TMAZ extrudes a greater volume of material on the RS into the SZ, particularly within the shoulder-affected zone.

Significantly, we observed clear boundaries between the SZ and TMAZ of the RS, in contrast to the indistinct boundaries observed in the AS. This distinction may be due to differences in how plasticized material flows on either side [64,65].

Grain structure and precipitate formation are major contributors to the mechanical anisotropy observed in Al-Li alloys. Variations in these factors can result in significant changes in hardness distribution and influence the overall mechanical properties of FSW 2060-T8 joints [66]. The size of recrystallized grains is closely tied to factors such as heat input and strain rate during welding. Additionally, temperature evolution during the FSW process plays a critical role in determining the microstructural characteristics of the welded joints [43].

During the FSW process, the SZ experiences intense deformation and dynamic recrystallization, leading to the nucleation and growth of new grains due to the thermal cycle. These grains initially bend and elongate under the strain. As the strain increases further, the elongated grains break down, forming equiaxed and more refined grains compared to the other FSW zones. The friction from the tool shoulder influences grain deformation in TMAZ. This friction causes the grains to tilt and elongate, forming an elliptical shape. The combined effects of heat generation and shear forces, which resist the flow of material, strongly correlate with this deformation. Conversely, the thermal and mechanical effects do not significantly alter the grain morphology between HAZ and BM [30]. As a result, the SZ is fully recrystallized, the TMAZ is partially recrystallized, and the HAZ exhibits grain coarsening without any recrystallization [65].

**Fig. 10:**
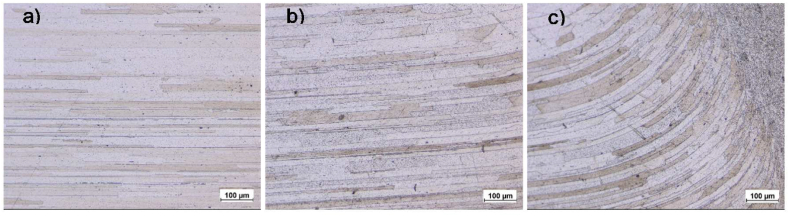
The OM micrograph of sample 1200-2-5 a) BM, b) HAZ, c) TMAZ.

Fig. 10 depicts the OM micrographs of sample 1200-2-5, highlighting the BM, HAZ, and TMAZ. The BM exhibits elongated grains, a characteristic result of the rolling process. Due to minimal heat input, the microstructure and mechanical properties of the BM remain largely unchanged [67]. These grains typically measure approximately 300–400 μm in length and 20–30 μm in width (Fig. 10-a). In the HAZ, the grains appear slightly coarsened (Fig. 10-b), primarily influenced by the welding cycle, which predominantly involves recovery processes [68]. In precipitation-hardened Al alloys, joint failure frequently occurs in the HAZ due to the coarsening and dissolution of precipitates, along with the formation of coarse grain boundary phases and precipitate-free zones [69].

In the TMAZ, the grains are observed to be longer and thinner compared to those in the BM (Fig. 10-c). which is attributed to This is attributed to relatively lower heat input and the plastic deformation occurring in this zone, where heavily distorted lamellar grains and finely equiaxed grains develop, reflecting insufficient recrystallization behavior. The precipitates in the TMAZ undergo a complex precipitation process involving dissolution, coarsening, and re-precipitation [70].

**Fig. 11:**
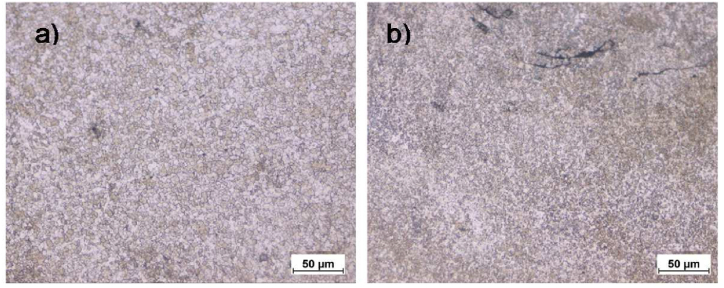
The SZ at a) 1200-2-5 and b) 1600-2-6.

In the SZ, dynamic recrystallization occurs due to the combined effects of forging force and heat generated by the tool shoulder. The rotating tool pin stirs the weld material, facilitating mixing and joint formation. As the tool advances, material flows behind the FSW tool pin, and the stirring action induces dynamic recrystallization due to severe plastic deformation. This results in the formation of fine, equiaxed grains in the stir region, accompanied by the dissolution of precipitates [71]. Fig. 11-a illustrates the SZ microstructure of sample 1200-2-5, while Fig. 11-b highlights the SZ microstructure of sample 1600-2-6.

The grains in the SZ of sample 1200-2-5 measured 5.9 μm, while those in sample 1600-2-6 were larger, measuring 7.7 μm. This difference in grain size accounts for the variation in microhardness values observed between the two samples. The increased heat input resulting from prolonged stirring time and elevated frictional heat in sample 1600-2-6 contributes to the larger grain sizes compared to those in sample 1200-2-5.

## 5 Conclusions

This study successfully applied the FSW process to join 2060-T8E30 Al-Li alloys using varied welding parameters determined through the Taguchi method. Surface integrity, microstructural characteristics, and mechanical properties comprehensively evaluated the welded joints' quality, leading to the following key findings.

1. The analysis of the S/N ratios revealed that rotational speed and axial force were the most influential factors affecting weld quality, ranking 1st and 2nd, respectively, followed by tool traverse speed. Precise control of these parameters is crucial for achieving optimal microstructural and mechanical properties.
2. Axial force significantly influences the characteristics of welded joints and should therefore be included in future research.
3. For the three parameters, the microhardness exhibited a similar trend, reaching its peak at the intermediate level of these parameters before decreasing at the highest levels. However, for TTS, a slight change in the microhardness values was observed.
4. The Taguchi technique identified the optimal welding parameters as TRS of 1200 rpm, TTS of 3 mm/s, and AF of 5 kN.
5. The refined grain structure and its uniform distribution are primarily responsible for the observed increase in microhardness within the weld zone of Al-Li 2060-T8E30.
6. Surface roughness measurements indicated minimal defects in the joints. The values recorded for defect-free joints were 3.05 μm for samples welded at 1200 rpm, 2 mm/s, and 5 kN, and 3.536 μm for samples welded at 1200 rpm, 3 mm/s, and 6 kN, respectively.
7. FSW has emerged as a cost-effective and versatile welding method that is widely applicable in the aerospace, automotive, and marine industries.

## CRediT authorship contribution statement

**Noah E. El-Zathry:** Writing – original draft, Methodology, Formal analysis. **Stephen Akinlabi:** Writing – review & editing, Supervision. **Wai Lok Woo:** Supervision. **Vivek Patel:** Writing – review & editing, Supervision, Methodology. **Rasheedat M. Mahamood:** Writing – original draft, Supervision.

## Funding

The authors thank the Department of Mechanical and Construction Engineering, Northumbria University, UK, for workspace and research facilities, and University West, Sweden, for access to the Friction Stir Welding (FSW) laboratory supporting this study.

## Data availability statement

No additional data was used for the research described in the article.

## Declaration of competing interest

The authors declare that they have no known competing financial interests or personal relationships that could have appeared to influence the work reported in this paper.
